# THE ADULT DAY CENTER: UNTAPPED TERRITORY FOR AGING RESEARCH

**DOI:** 10.1093/geroni/igad104.0099

**Published:** 2023-12-21

**Authors:** Tina Sadarangani, Fei Sun

## Abstract

Adult day service centers (ADCs) in the United States are a vital, but overlooked source of community-based long-term care for diverse older adults with multiple chronic conditions, including dementia. The National Institute on Aging has called for more research on ADCs. In this interdisciplinary symposium, we present challenges and opportunities in conducting research in these congregate settings. First, we look at the operating status of ADCs nationally in the aftermath of the COVID-19 pandemic and disparities in access to care using longitudinal data from the National Post-acute and Long-term Care Study. Then we examine the lack of electronic health record utilization and clinical data capture in 4,035 ADCs nationally and discuss strategies to modernize technology in these settings to facilitate data collection. We subsequently present a new database, developed vis a vis an academic/community partnership between the National Adult Day Services Association and researchers, that centralizes and makes data collection feasible in ADCs with limited resources. We will also explore factors supporting intervention research that are unique to ADCs. Finally, we present a protocol adapted for the ADC setting, to test a mobile application that facilitates data collection and enables communication between ADCs, caregivers, and healthcare providers. Our findings show that, while research in ADCs has been limited historically, academic, government, and community partners are working together to spur pragmatic innovations that modernize these settings and better resource them to elevate standards of care.

